# Targeted overexpression of the long noncoding RNA ODSM can regulate osteoblast function in vitro and in vivo

**DOI:** 10.1038/s41419-020-2325-3

**Published:** 2020-02-18

**Authors:** Yixuan Wang, Ke Wang, Lijun Zhang, Yingjun Tan, Zebing Hu, Lei Dang, Hua Zhou, Gaozhi Li, Han Wang, Shu Zhang, Fei Shi, Xinsheng Cao, Ge Zhang

**Affiliations:** 1 The Key Laboratory of Aerospace Medicine, Ministry of Education, Air Force Medical University, Xi’an, 710032 Shaanxi China; 2 State Key Laboratory of Space Medicine Fundamentals and Application, China Astronaut Research and Training Center, Beijing, 100094 China; 3 Institute for Advancing Translational Medicine in Bone & Joint Diseases, School of Chinese Medicine, Hong Kong Baptist University, Hong Kong SAR, China

**Keywords:** Molecular biology, Diseases

## Abstract

Ameliorating bone loss caused by mechanical unloading is a substantial clinical challenge, and the role of noncoding RNAs in this process has attracted increasing attention. In this study, we found that the long noncoding RNA osteoblast differentiation-related lncRNA under simulated microgravity (lncRNA ODSM) could inhibit osteoblast apoptosis and promote osteoblast mineralization in vitro. The increased expression level of the lncRNA ODSM partially reduced apoptosis and promoted differentiation in MC3T3-E1 cells under microgravity unloading conditions, and the effect was partially dependent on miR-139-3p. LncRNA ODSM supplementation in hindlimb-unloaded mice caused a decrease in the number of apoptotic cells in bone tissue and an increase in osteoblast activity. Furthermore, targeted overexpression of the lncRNA ODSM in osteoblasts partially reversed bone loss induced by mechanical unloading at the microstructural and biomechanical levels. These findings are the first to suggest the potential value of the lncRNA ODSM in osteoporosis therapy and the treatment of pathological osteopenia.

## Introduction

Bone is a dynamic tissue that is constantly resorbed by osteoclasts and reconstructed by osteoblasts^1,2^. Osteoporosis is associated with a number of stimuli, including hormone fluctuations, nutrition, and inflammatory and mechanical loading^3–5^. Mechanical loads have been considered the basis for the normal development and maintenance of the musculoskeletal system. Long-term bed rest due to spinal injury or other injuries, as well as the reduction in bone loading caused by the microgravity (MG) environment during space flight, results in bone loss^5–7^. Hindlimb-unloaded (HU) animal models are the most commonly used in vivo models for inducing bone loss due to unloading^8,9^. Osteopenia is caused by an imbalance of bone resorption and formation, while impaired osteoblast function is a main cause of bone loss due to unloading^10,11^. Clinostats, random positioning machines, or rotary wall vessels are usually used to study cell responses to conditions lacking mechanical loading^12–14^. It is necessary and feasible to further study the molecular mechanisms regulating osteoblast function in the unloading environment and to subsequently develop a promising strategy for bone formation.

Long noncoding RNAs (lncRNAs) are a class of RNAs with a length of >200 nucleotides. lncRNAs do not encode proteins but engage in numerous important physiological phenomena and pathological processes^15,16^. Recent studies have indicated that lncRNAs are associated with osteoporosis and other bone diseases^17^. Inhibiting AK016739 expression in vivo promoted osteogenic gene expression and rescued calvarial bone formation in ovariectomy (OVX) mice^18^. LncRNAs are involved in osteoblast differentiation and apoptosis. The subcellular localization of lncRNA determines its potential modes of action in osteoblasts. Nuclear lncRNAs are functionally implicated in gene regulatory processes, such as activation of transcription and epigenetic gene regulation. For example, lncRNA Bmncr could serve as a scaffold to facilitate the interaction of TAZ and ABL, and thus facilitated the assembly of the TAZ and RUNX2/PPARG transcriptional complex, which enhanced osteogenesis^19^. LncRNA ODIR1 inhibited the osteogenic differentiation of human mesenchymal stem cells through the FBXO25/H2BK120ub/H3K4me3/OSX axis^20^. LncRNAs localized in the cytoplasm are shown to be involved in posttranscriptional gene regulatory processes, including acting as competitive endogenous RNAs (ceRNAs) to combine with microRNAs (miRNAs) and impair miRNA activity through upregulating miRNA target gene expression^21,22^. For example, the lncRNA PGC1β-OT1 regulated osteogenic differentiation through antagonizing miR-148a-3p (ref. ^23^). In human bone-marrow-derived mesenchymal stem cells, linc-ROR functioned as a miRNA sponge for miR-138 and miR-145 to promote osteogenic differentiation^24^. Overexpression of lncRNA-ORLNC1 resulted in osteoporosis and impaired osteogenic capacity in an OVX mouse model of osteoporosis^25^. Additionally, the lncRNA TSIX promoted osteoblast apoptosis by downregulating miR-30a-5p (ref. ^26^). Our previous study found that the lncRNA NONMMUT002009 [osteoblast differentiation-related lncRNA under simulated microgravity (lncRNA ODSM)] interacted with miR-139-3p and was able to regulate the osteoblast differentiation. MiR-139-3p alleviated the effect of MG unloading on differentiation and apoptosis in MC3T3-E1 cells, through its target gene ELK1 (ref. ^27^). Based on the above results, we aimed to verify whether intervening with lncRNA ODSM expression can affect osteoblast functions and bone formation in a mechanical unloading environment in vitro or in vivo.

With an increasing number of noncoding RNAs found to regulate many pathological processes, the value of miRNAs and lncRNAs as therapeutic targets in many diseases has received increasing attention^28–30^. Intervention with the expression of miRNAs or lncRNAs in vivo also had an impressive therapeutic effect in the diseases caused by mechanical unloading. For example, skeletal muscle-specific overexpression of lncMUMA attenuated and reversed the decrease of the muscle mass, structure and function of 42-day HU mice^28^. Enforced expression of the lncRNA MAR1 reversed muscle atrophy in a mouse model of hindlimb suspension^29^. For bone loss and bone anabolic therapy, (AspSerSer)_6_-liposomes are a promising targeted delivery system for carrying RNA specifically to bone formation surfaces^31^. Inhibiting miR-214 or increasing miR-33-5p expression via targeted delivery by (AspSerSer)_6_-liposomes counteracted bone loss in the HU mouse model^30,32^. However, whether targeted intervention with lncRNA expression—especially that of the lncRNA ODSM—in the osteogenic region can improve bone loss after mechanical unloading still needs to be explored.

In this study, we found that the lncRNA ODSM could inhibit osteoblast apoptosis and promote osteoblast mineralization. The lncRNA ODSM could regulate ELK1 expression by interacting with miR-139-3p. The increased expression level of the lncRNA ODSM partly reduced apoptosis and promoted differentiation in MC3T3-E1 cells in a manner partially dependent on miR-139-3p in a MG unloading environment. Furthermore, we provided the first confirmation of the anti-osteoporotic effect of the lncRNA ODSM by the (AspSerSer)_6_-liposome delivery system in a mouse model of HU.

## Results

### LncRNA ODSM inhibits osteoblast apoptosis and promotes osteoblast mineralization

**Fig. 1:**
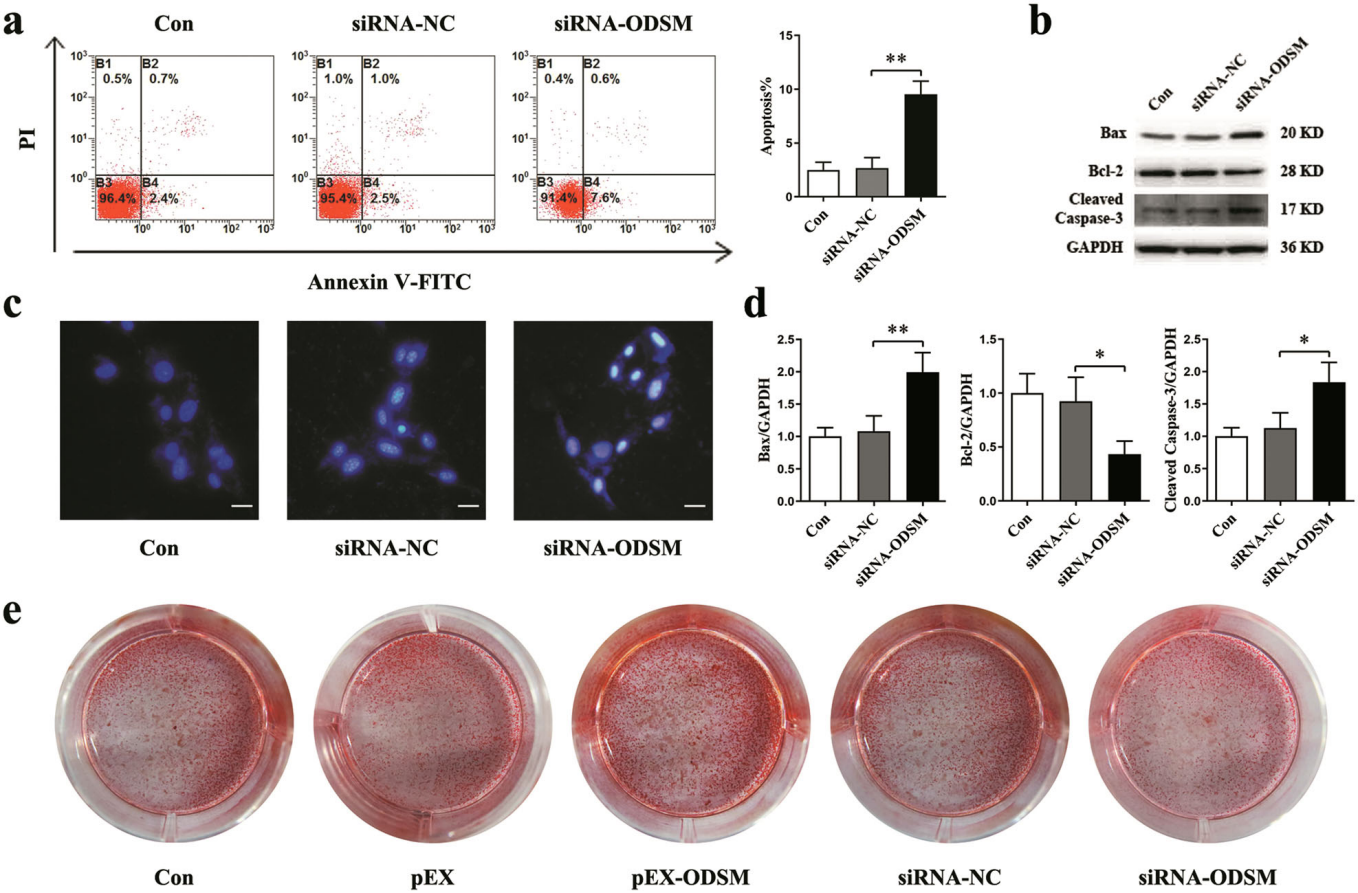
LncRNA ODSM inhibits osteoblast apoptosis and promotes osteoblast mineralization. siRNA-ODSM, pEX-ODSM, and their corresponding negative controls were transfected into MC3T3-E1 cells. **a** Flow cytometric analysis of apoptosis in osteoblasts stained with Annexin V-FITC/PI (*N* = 3). **b**, **d** Protein levels of Bax, Bcl-2, and cleaved caspase-3 in osteoblasts (*N* = 3). **c** Representative images of Hoechst 33258 staining in osteoblasts (*N* = 3). Scale bar, 50 µm. **e** Representative images of Alizarin red staining in osteoblasts (*N* = 3). *P < 0.05, **P *<* 0.01 vs. the negative control.

The lncRNA ODSM expression level was much higher in the femurs than in other tissues and organs in mice, and the level of the lncRNA ODSM was significantly decreased in the femurs of HU mice (Supplementary Fig. 1). To further explore the biological effects of the lncRNA ODSM on osteoblast function in vitro, we used a RNA interference and overexpression vector to change the expression of the lncRNA ODSM in MC3T3-E1 cells. Silencing the lncRNA ODSM markedly increased the ratio of apoptotic osteoblasts in the small interfering RNA (siRNA)-ODSM group (Fig. 1a). The levels of the apoptosis-related protein Bax and cleaved caspase-3 in the siRNA-ODSM group were increased, while the protein level of Bcl-2 was significantly decreased (Fig. 1b, d). Hoechst 33258 staining was used to evaluate the apoptotic status of MC3T3-E1 cells. Blue apoptotic nuclei were observed in siRNA-ODSM-treated cells (Fig. 1c). To observe the mineralization of osteoblasts, alizarin red staining was used. There was more mineral deposition in the pEX-ODSM groups and less mineral deposition in the siRNA-ODSM-treated cells than in the control or negative control (NC) groups (Fig. 1e).

### LncRNA ODSM interacts with miR-139-3p to regulate ELK1 expression

**Fig. 2:**
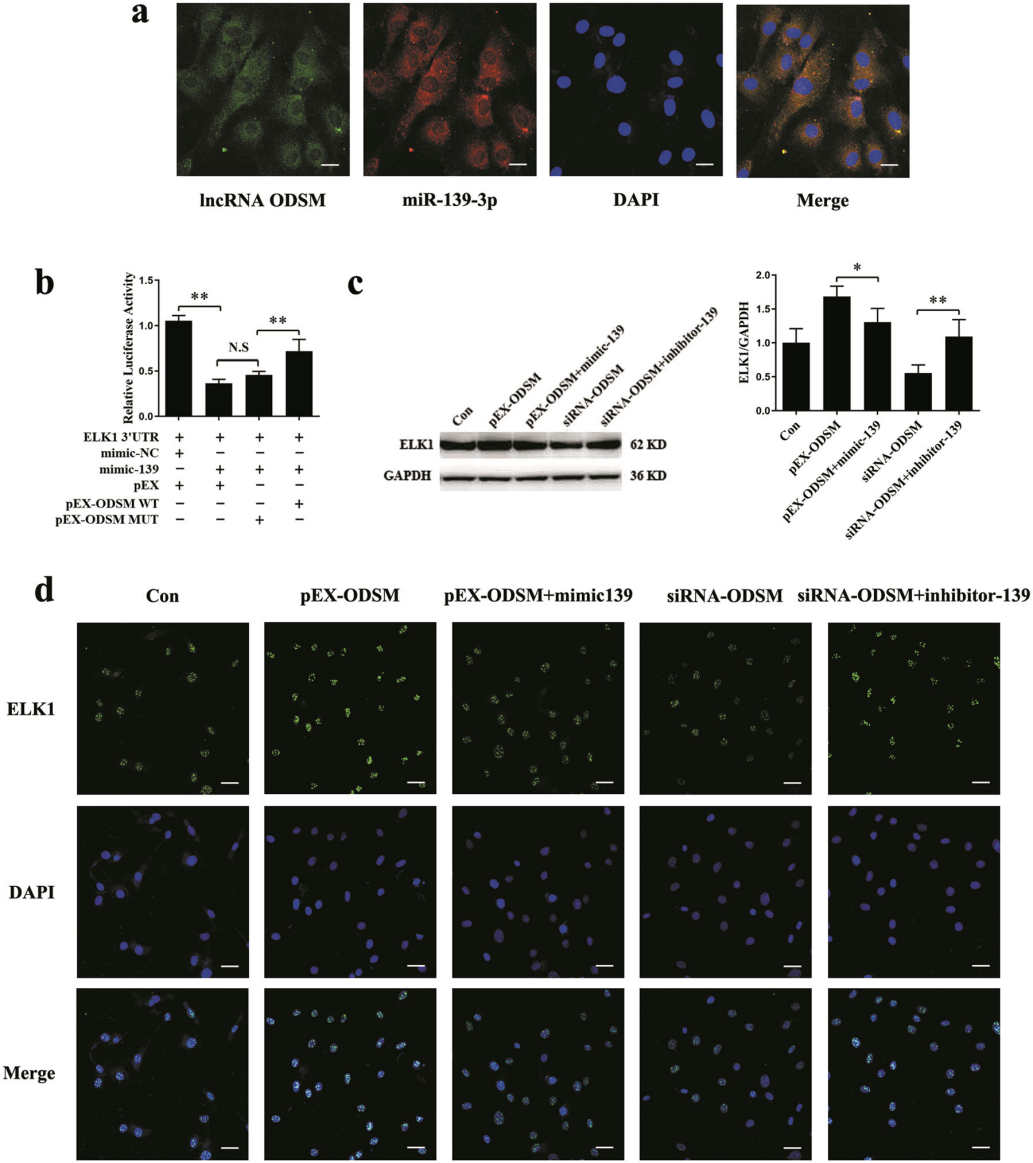
LncRNA ODSM interacts with miR-139-3p to regulate ELK1 expression. **a** The co-localization of lncRNA ODSM and miR-139-3p in MC3T3-E1 cells determined by RNA fluorescence in situ hybridization (*N* = 3). Scale bar, 100 µm. **b** Luciferase activity in 293 T cells transfected with luciferase reporter vectors containing the ELK1 3′-UTR and treated with mimic-139, pEX-ODSM WT, pEX-ODSM MUT, or the corresponding controls for 24 h (*N* = 3). **c** Protein levels of ELK1 in osteoblasts (*N* = 3). **d** Immunostaining analysis of the expression levels of ELK1 in osteoblasts (*N* = 3). Scale bar, 50 µm. *P *<* 0.05, **P *<* 0.01 vs. the negative control.

Our previous experiments confirmed that lncRNA ODSM and miR-139-3p interact with and repress each other^27^. By using the online lncRNA location prediction software lncLocator (http://www.csbio.sjtu.edu.cn/bioinf/lncLocator/) and cell fractionation followed by quantitative reverse transcription polymerase chain reaction (qRT-PCR), we identified that lncRNA ODSM expression was located in the cytoplasm and nuclei of cells (Supplementary Fig. 2). To further confirm the interaction of lncRNA ODSM and miR-139-3p, we performed RNA fluorescence in situ hybridization (RNA-FISH) in MC3T3-E1 cells. The data showed the co-localization of lncRNA ODSM and miR-139-3p in the cytoplasm (Fig. 2a). Western blotting analyses showed that the protein translation of ELK1 was suppressed by lncRNA ODSM, and this effect had little influence on ELK1 phosphorylation (Supplementary Fig. 3). Thus, further study was conducted to explore whether the lncRNA ODSM can regulate the expression of the miR-139-3p-targeted gene ELK1 in MC3T3-E1 cells. The regulation of ELK1 by miR-139-3p relies on the 3′-UTR region of ELK1, so if the regulatory effect of the lncRNA ODSM on ELK1 depends on competitive binding to miR-139-3p, then the lncRNA ODSM should also have a regulatory effect on the 3′-UTR region of ELK1. We constructed luciferase reporter vectors containing the ELK1 3′-UTR sequence and the lncRNA ODSM sequence with a wild-type (pEX-ODSM WT) or mutated miR-139-3p binding site (pEX-ODSM MUT) as previously reported^27^, and we then transfected these vectors into cells. Overexpression of the lncRNA ODSM, but not pEX-ODSM MUT, partly blocked the mimic-139-induced reduction in luciferase activity of the ELK1 reporter vector. The results suggested that the upregulation of the lncRNA ODSM increased the luciferase activity of the ELK1 3′-UTR reporter by competitively binding miR-139-3p (Fig. 2b). To further investigate whether the lncRNA ODSM regulates ELK1 protein expression partially through interacting with miR-139-3p, we simultaneously regulated the expression of the lncRNA ODSM and miR-139-3p in MC3T3-E1 cells. Co-transfection of mimic-139 with pEX-ODSM partially inhibited the pEX-ODSM-induced increase in ELK1 expression, whereas treatment with inhibitor-139 partially blocked the siRNA-ODSM-induced reduction in ELK1 expression, as indicated by western blot and indirect immunofluorescence assays (Fig. 2c, d). Similarly, co-transfection pEX-ODSM with mimic-139 significantly attenuated the decrease in the expression level of ELK1 in the mimic-139 group. Meanwhile co-transfection siRNA-ODSM with inhibitor-139, the expression of ELK1 markedly decreased compared with that in the inhibitor-139 group (Supplementary Fig. 4).

### Overexpression of the lncRNA ODSM partially reduces apoptosis and promotes differentiation in MC3T3-E1 cells under MG unloading conditions

**Fig. 3:**
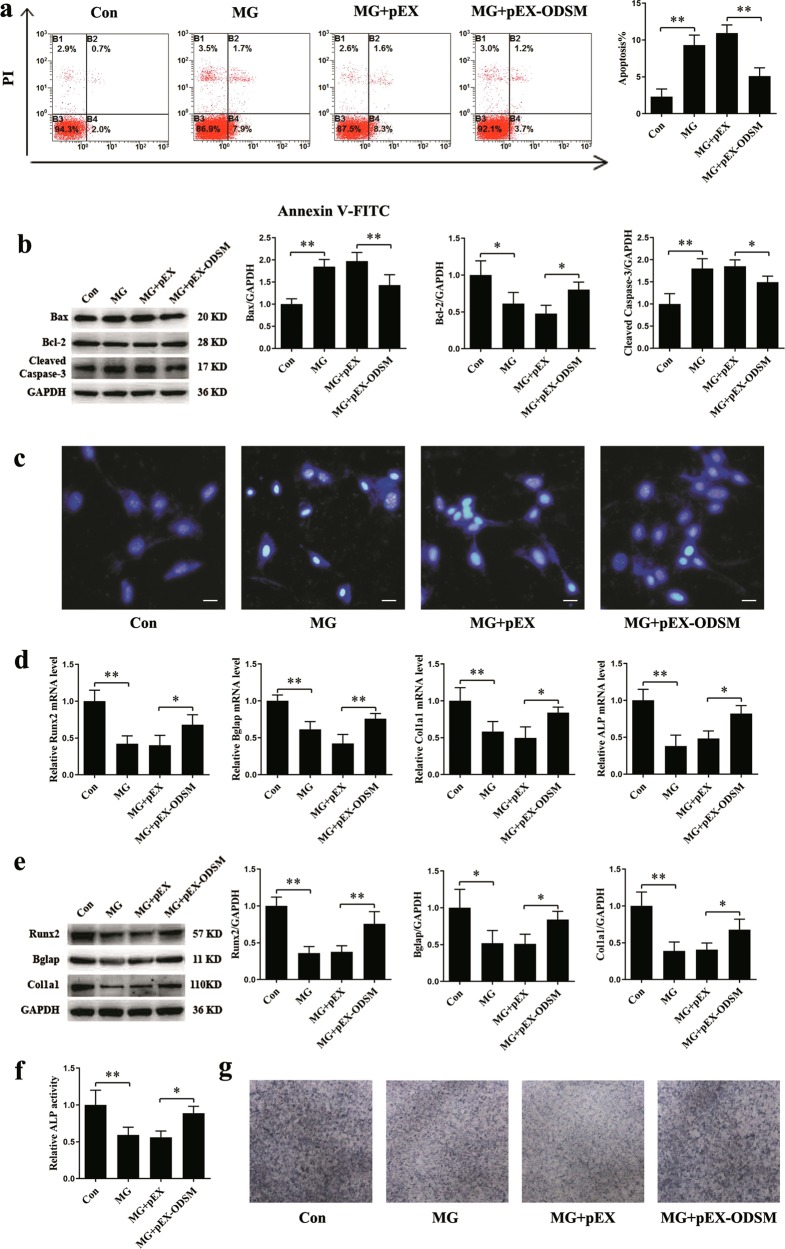
Overexpression of the lncRNA ODSM partially reduces apoptosis and promotes differentiation in MC3T3-E1 cells under MG unloading conditions. **a** Flow cytometric analysis of apoptosis in osteoblasts stained with Annexin V-FITC/PI (*N* = 3). **b** Protein levels of Bax, Bcl-2, and cleaved caspase-3 in osteoblasts (*N* = 3). **c** Representative images of Hoechst 33258 staining in osteoblasts (*N* = 3). Scale bar, 50 µm. **d** mRNA expression levels of osteoblast marker genes (Runx2, Bglap, Col1a1, and ALP; normalized to GAPDH) in osteoblasts treated with pEX-ODSM or the corresponding control (*N* = 3). **e** Protein levels of Runx2, Bglap, and Col1a1 in osteoblasts (*N* = 3). **f** ALP activity analysis in osteoblasts at 48 h (*N* = 3). **g** Representative images of ALP staining in osteoblasts (*N* = 3). *P *<* 0.05, **P *<* 0.01 vs. the control or negative control.

Experiments have shown that mechanical unloading can increase the apoptosis and decrease the differentiation of osteoblasts^27^. To investigate whether the lncRNA ODSM can affect the differentiation of osteoblasts in a MG unloading environment, MC3T3-E1 cells were transfected with pEX-ODSM for 12 h and were then cultured in a MG unloading environment for 48 h. In the pEX-ODSM group, the ratio of apoptotic MC3T3-E1 cells was markedly decreased (Fig. 3a). Compared with MC3T3-E1 cells in the MG + pEX group, cells in the MG + pEX-ODSM group exhibited the decreased expression levels of the apoptosis-related proteins Bax and cleaved caspase-3 but increased Bcl-2 protein expression levels (Fig. 3b). Hoechst 33258 staining also showed a marked decrease in blue apoptotic nuclei in the pEX-ODSM group in the MG unloading environment (Fig. 3c).

The lncRNA ODSM is closely related to osteoblast differentiation, but whether it can affect osteoblast differentiation in a MG unloading environment remains to be studied. pEX-ODSM transfection significantly upregulated the expression of the osteogenic genes Runx2, Bglap, Col1a1, and alkaline phosphatase (ALP) in the MG unloading environment (Fig. 3d). The protein levels of Runx2, Bglap, and Col1a1 were also increased in the MG + pEX-ODSM groups (Fig. 3e). ALP activity assays and ALP staining showed similar trends (Fig. 3f, g).

### LncRNA ODSM-mediated regulation of osteoblast apoptosis and differentiation partially depends on miR-139-3p under MG unloading conditions

**Fig. 4:**
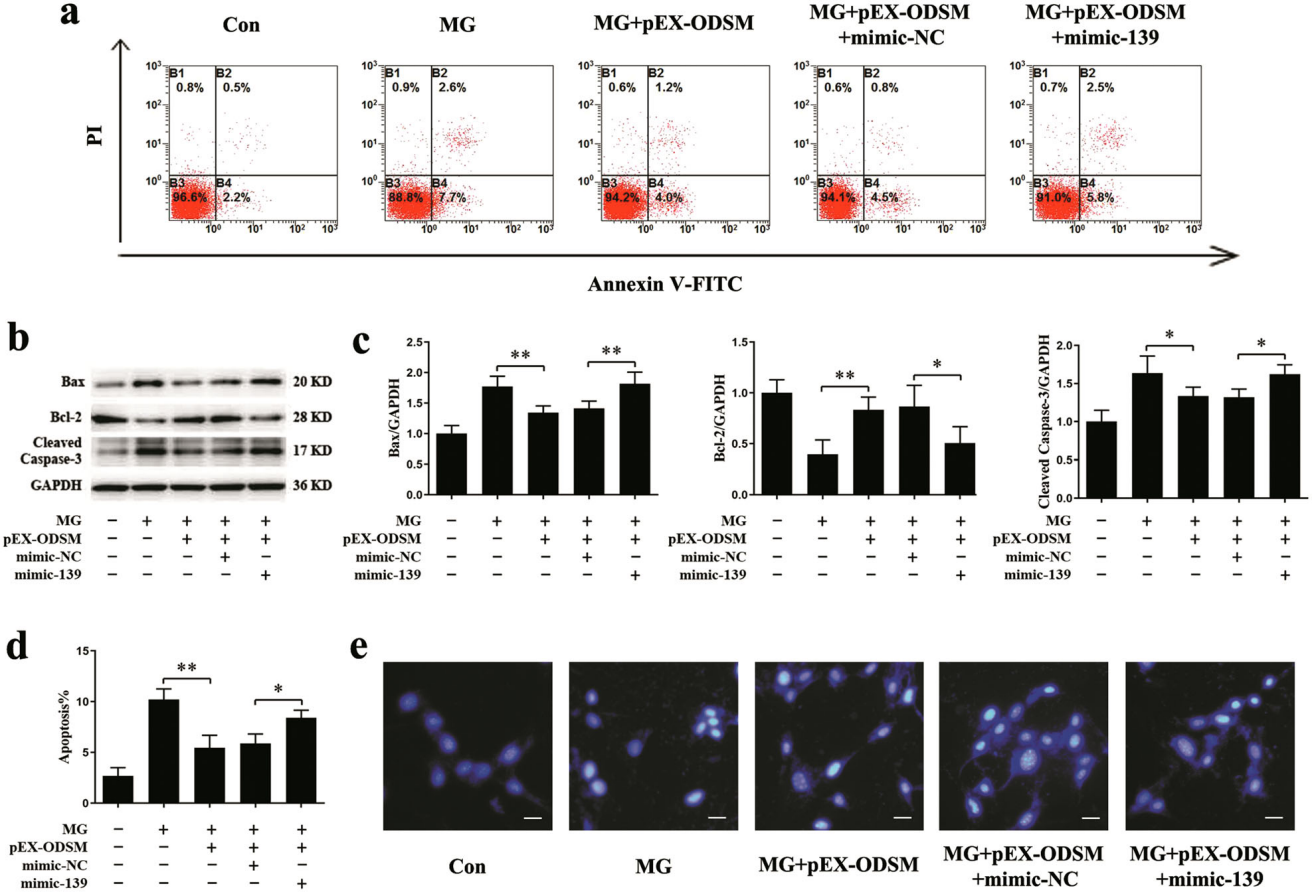
LncRNA ODSM-mediated regulation of osteoblast apoptosis partially depends on miR-139-3p under MG unloading conditions. pEX-ODSM and mimic-139 were cotransfected into MC3T3-E1 cells and then cultured in a MG unloading environment for 48 h. **a**, **d** Flow cytometric analysis of apoptosis in osteoblasts stained with Annexin V-FITC/PI (*N* = 3). **b**, **c** Protein levels of Bax, Bcl-2, and cleaved caspase-3 in osteoblasts (*N* = 3). **e** Representative images of Hoechst 33258 staining in osteoblasts (*N* = 3). Scale bar, 50 µm. *P *<* 0.05, **P *<* 0.01 vs. the control or negative control.

To further investigate the mechanism by which the lncRNA ODSM regulates the osteoblast apoptosis and differentiation in a MG unloading environment, pEX-ODSM and mimic-139 or its negative control were cotransfected into MC3T3-E1 cells for 12 h, and cells were then cultured in a MG unloading environment for 48 h. The ratio of apoptotic osteoblasts was markedly reversed in MC3T3-E1 cells treated with pEX-ODSM in the MG unloading environment, and apoptosis was abolished by overexpression of miR-139-3p (Fig. 4a, d). Mimic-139 significantly attenuated the decrease in the Bax and cleaved caspase-3 protein levels, and the increase in the Bcl-2 protein level induced by pEX-ODSM in the MG unloading environment (Fig. 4b, c). Additionally, Hoechst 33258 staining showed that the lncRNA ODSM reduced the number of blue apoptotic nuclei in the pEX-ODSM group, but this decrease was diminished by mimic-139 in the MG unloading environment (Fig. 4e).

**Fig. 5:**
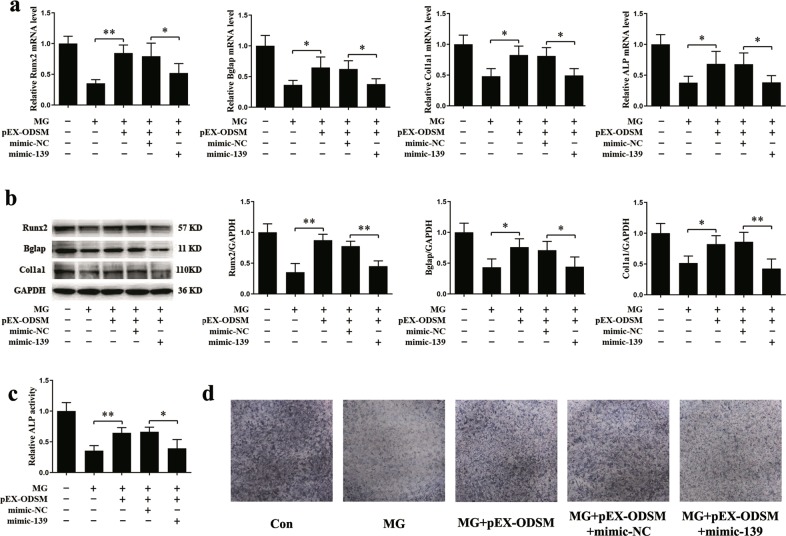
LncRNA ODSM-mediated regulation of osteoblast differentiation partially depends on miR-139-3p under MG unloading conditions. pEX-ODSM and mimic-139 were cotransfected into MC3T3-E1 cells and then cultured in a MG unloading environment for 48 h. **a** qRT-PCR analysis of osteoblast marker gene (Runx2, Bglap, Col1a1, and ALP; normalized to GAPDH) mRNA levels in osteoblasts (*N* = 3). **b** Western blot analysis of Runx2, Bglap, and Col1a1 protein expression in osteoblasts (*N* = 3). **c** ALP activity analysis in osteoblasts at 48 h (*N* = 3). **d** Representative images of ALP staining in osteoblasts (*N* = 3). *P *<* 0.05, **P *<* 0.01 vs. the negative control.

Osteogenic genes, such as Runx2, Bglap, Col1a1, and ALP were upregulated by pEX-ODSM, whereas miR-139-3p overexpression reduced the upregulation induced by the lncRNA ODSM in the MG unloading environment (Fig. 5a). The changes in the Runx2, Bglap, and Col1a1 protein levels, ALP activity and ALP expression also showed similar trends (Fig. 5b–d). These results confirmed that lncRNA ODSM suppressed osteoblast apoptosis and increased the osteogenic differentiation by interacting with miR-139-3p.

### LncRNA ODSM partially counteracted the decrease in osteoblast activity and bone formation in HU mice

**Fig. 6:**
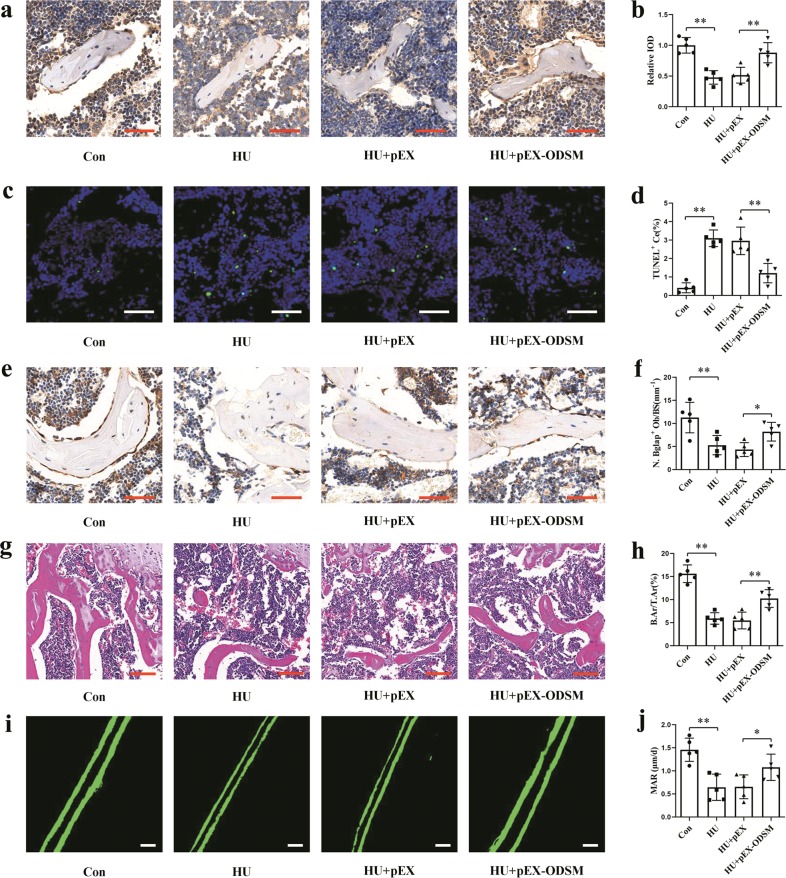
LncRNA ODSM partially counteracted the decreases in osteoblast activity and bone formation in HU mice. **a** Representative images of ELK1 staining in the distal femurs of mice in each indicated group. Scale bar, 50 µm. **b** Statistical analysis of the relative integrated optical density (IOD) values of femurs (*N* = 5). **c** Representative TUNEL images of the distal femurs of mice in each indicated group. Scale bar, 50 µm. **d** Statistical analysis of TUNEL-positive cell percentages (*N* *=* 5). **e** Representative images of Bglap staining of the distal femurs of mice in each indicated group. Scale bar, 50 µm. **f** Statistical analysis of the number of positive osteoblasts per bone surface in the femurs (N. Bglap^+^ Ob/BS; *N* = 5). **g** Representative images of H&E staining of the distal femurs of mice in each indicated group. Scale bar, 100 µm. **h** Statistical analysis of the histological parameter bone area/total area (B.Ar/T.Ar) in the proximal region of different groups via H&E staining (*N* = 5). **i** Representative images of new bone formation assessed by double calcein labeling. Scale bar, 20 µm. **j** Statistical analysis of the histological parameter MAR in each indicated group (*N* = 5). *P *<* 0.05, **P *<* 0.01 vs. the control or negative control.

To investigate the role of the lncRNA ODSM in vivo, we used the (AspSerSer)_6_-liposome system to deliver the lncRNA ODSM to the bone formation zone. Before the hindlimb unloading procedure, mice in the experimental groups were injected with three consecutive injections of HU + pEX and HU + pEX-ODSM liposomes via the caudal vein. The expression of the lncRNA ODSM was upregulated, and the expression of miR-139-3p was downregulated in the femurs of HU mice treated with pEX-ODSM relative to these levels in the HU + pEX group on the 21th day of hindlimb unloading (Supplementary Fig. 5). After 21 days of hindlimb unloading, ELK1 immunohistochemical staining showed that ELK1 expression was significantly decreased in the HU group, whereas this decrease was reversed in the HU + pEX-ODSM group (Fig. 6a, b). The proportions of TUNEL-positive apoptotic cells were significantly higher in the distal femurs of HU mice than in those of Con mice, and fewer TUNEL-positive apoptotic cells were seen in HU + pEX-ODSM group mice than in HU + pEX group mice (Fig. 6c, d). The Bglap staining results showed that the numbers of Bglap^+^ osteoblasts were significantly decreased in the femurs of HU mice. There were more Bglap^+^ osteoblasts in HU + pEX-ODSM group mice than in HU + pEX group mice (Fig. 6e, f). The hematoxylin and eosin (H&E) staining results showed that compared to control mice, HU mice exhibited a reduction in the bone area/total area ratio (B.Ar/T.Ar). However, administration of pEX-ODSM significantly restored the B.Ar/T.Ar (Fig. 6g, h). Calcein double labeling indicated that bone formation was significantly decreased in HU group mice compared with that in Con group mice, and that bone formation was increased in pEX-ODSM-treated HU group mice compared to that in negative control mice. Regarding bone histomorphometric parameters, the mineral apposition rate (MAR) showed the same trends (Fig. 6i, j).

### Enforced lncRNA ODSM expression attenuated the adverse effects on bone architecture and mechanical properties in HU mice

**Fig. 7:**
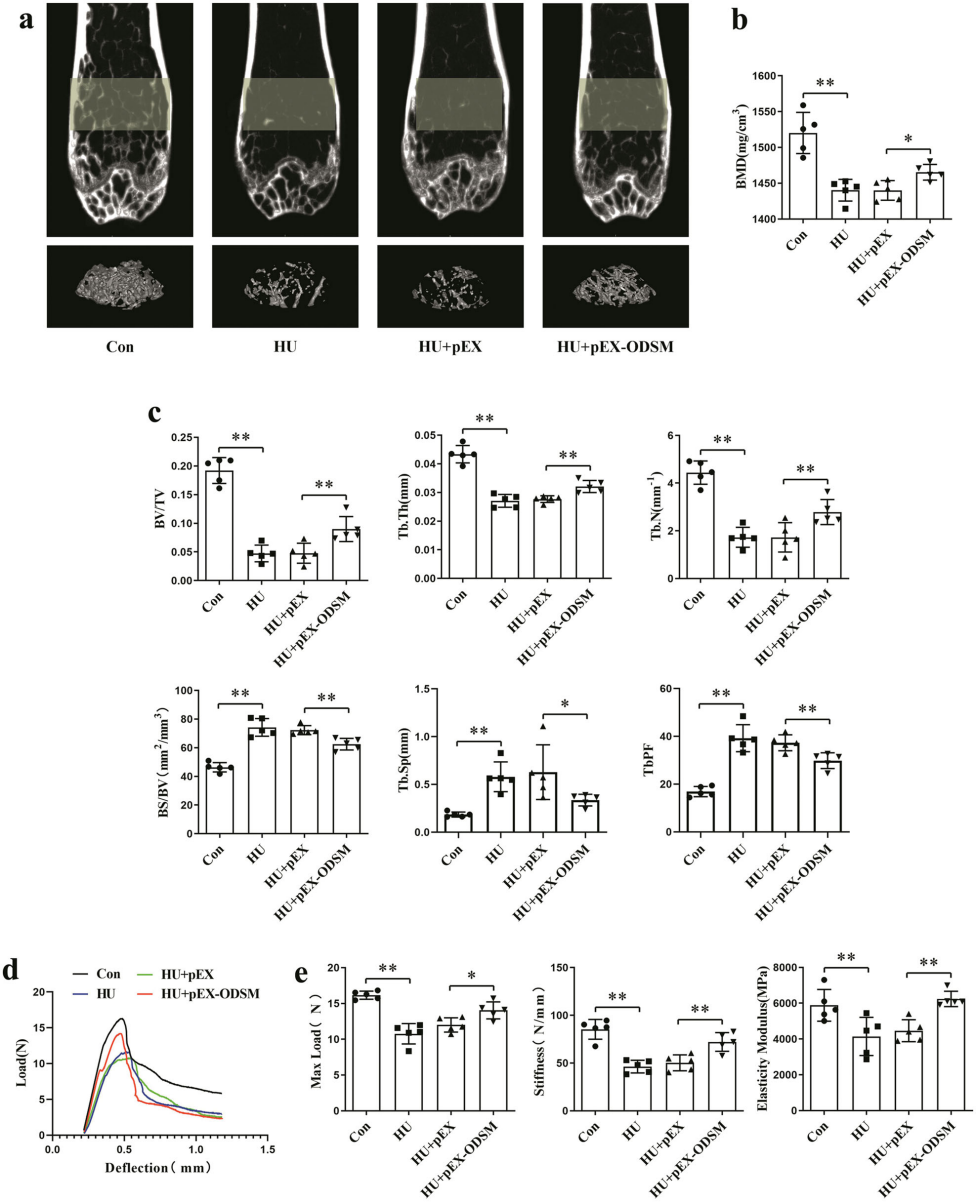
Enforced lncRNA ODSM expression attenuated the adverse effects on bone architecture and mechanical properties in HU mice. **a** Representative images acquired by microCT examination of the trabecular architecture of the distal femurs of mice in each group. **b**, **c** microCT analysis of the ROI in the distal femurs of mice in each group. The three-dimensional indexes were bone mineral density (BMD), relative bone volume (BV/TV), trabecular bone thickness (Tb.Th), trabecular bone number (Tb.N), bone surface to bone volume ratio (BS/BV), trabecular bone separation (Tb.Sp), and trabecular bone pattern factor (TbPF; *N* *=* 5). **d** Representative load-displacement curves of mouse femurs from each group. **e** The biomechanical parameters (maximum load, stiffness, and modulus of elasticity) of femurs harvested from each group were analyzed (*N* *=* 5). *P *<* 0.05, **P *<* 0.01 vs. the control or negative control.

In addition to observing the effect of the lncRNA ODSM on new bone formation in HU mice, we further explored its effect on bone structure. After 21 days of hindlimb unloading, the two-dimensional (2D) and three-dimensional (3D) image reconstruction from microCT examination of the distal femurs of the mice showed that in the HU groups, the trabecular structures and bone mass in the HU groups were severely damaged, although these structures were more complete in the HU + pEX-ODSM group than in the HU + pEX group (Fig. 7a). MicroCT analysis showed significant decreases in the bone mineral density (BMD), relative bone volume (BV/TV), trabecular bone thickness (Tb.Th), and trabecular bone number (Tb.N) in HU mice, whereas the decreases in these parameters were reversed in HU + pEX-ODSM group mice. In addition, there were marked increases in the ratio of bone surface to bone volume (BS/BV), trabecular bone separation (Tb.Sp), and trabecular bone pattern factor (TbPF) in the HU group compared with those in the Con group, and these increases were attenuated by treatment with pEX-ODSM (Fig. 7b, c).

To further explore the strength and structural properties of the femurs, we used a three-point bending test, which causes a mid-diaphyseal fracture under controlled loading conditions. Typical load-displacement curves for each group were plotted according to the data (Fig. 7d). The structural parameters of the mouse femurs (including the maximum load, yield load, stiffness, and modulus of elasticity) were markedly decreased in the HU group compared to those in the Con group and were not significantly different from those in the HU + pEX group. pEX-ODSM overexpression in HU mice partially counteracted the decreases in these parameters in the HU + pEX group (Fig. 7e). These results indicated that the femurs of HU + pEX-ODSM mice were more resistant to fracture than those of HU + pEX mice.

## Discussion

An increasing number of experiments have confirmed that lncRNAs can regulate osteoblast functions, such as differentiation and apoptosis^18,23,26,33^. Our previous research first identified a sensitive lncRNA, lncRNA ODSM, in osteoblasts in a MG unloading environment that could promote osteoblast differentiation^27^. In this study, we further confirmed that the lncRNA ODSM can inhibit the apoptosis and promote the mineralization of MC3T3-E1 cells, and that it can regulate ELK1 expression in a manner partially dependent on miR-139-3p. Enforced expression of the lncRNA ODSM attenuated the increase in osteoblast apoptosis and the decrease in osteoblast differentiation in vitro. In addition, targeted overexpression of the lncRNA ODSM partly reversed bone loss following mechanical unloading in HU mice. The lncRNA ODSM could be a promising therapeutic target in osteoporosis induced by mechanical unloading.

LncRNAs containing miRNA binding sites could serve as molecular sponges to inhibit miRNA function^21,22^. In our previous study, we found that the lncRNA ODSM was mainly located in the cytoplasm of MC3T3-E1 cells and could regulate the expression of miR-139-3p. The luciferase assay and AGO2 immunoprecipitation (RIP) assay validated the direct binding of the lncRNA ODSM to miR-139-3p. We first identified that miR-139-3p can regulate osteoblast differentiation and apoptosis under both normal and MG unloading conditions^27^. Several studies have shown that miR-139-3p is associated with several cancers, such as myeloid leukemia, hepatocellular carcinoma, and bladder cancer^34–36^. MiR-139-5p, which originates from the opposite arm of the same miRNA precursor as miR-139-3p, can inhibit osteogenesis in mesenchymal stem cells^37^. One of the target genes of miR-139-3p is ELK1. ELK1, a transcription factor in the ETS family, acts as an essential component of the mitogen regulation pathway that activates the mitogen-activated protein kinase cascade^38^. It has been proven that ELK1 regulates various cell processes and is closely related to osteogenic differentiation^39–41^. We also found that ELK1 could promote the osteoblast differentiation and inhibit the osteoblast apoptosis, and was essential for the regulation of osteoblast differentiation and apoptosis by miR-139-3p^27^. Although ELK1 is a transcription factor, it is also regulated by miRNAs or lncRNAs^42,43^. The lncRNA TCONS_00026907 acts as a ceRNA to upregulate the ELK1 expression through inhibiting miR-143-5p in HeLa and SiHa cells^43^. It was observed in our study that the lncRNA ODSM regulated the expression of ELK1, which is the target gene of miR-139-3p. In addition, the regulatory effect of the lncRNA ODSM on osteoblast function depended partly on miR-139-3p under both normal and MG unloading conditions.

To better deliver lncRNA ODSM to osteoblasts in vivo, we used the (AspSerSer)_6_ delivery system, which can target bone formation surfaces. It has been proven that (AspSerSer)_6_ has an increased binding affinity to bone formation surfaces, which could reduce its delivery to nonskeletal organs^31^. Conjugation with (AspSerSer)_6_ promoted the binding of chalcone derivatives to targeted osteoblasts, thereby promoting systemic bone formation in BMP-2n/Smurf1e subgroup mice^44^. Administration of Plekho1 siRNA encapsulated in the osteoblast-specific delivery system reversed established osteoporosis during aging in mice^45^. Additionally, in our own previous experiments, it was confirmed that targeted replenishment of miR-33-5p with the (AspSerSer)_6_ delivery system attenuated the development of osteopenia induced by mechanical unloading in mice^32^. To further investigate the effects of lncRNAs on bone loss caused by mechanical unloading, we used the (AspSerSer)_6_ delivery system to accomplish a targeted increase in the lncRNA ODSM levels in bone formation surfaces. Although the lncRNA ODSM did not fully restore the bone loss caused by mechanical unloading in HU mice, overexpression of the lncRNA ODSM markedly alleviated the loss of bone mass and improved bone architecture and mechanical properties.

In conclusion, this study revealed that the lncRNA ODSM can inhibit osteoblast apoptosis and promote osteoblast mineralization in vitro. By interacting with miR-139-3p, lncRNA ODSM can regulate the expression of its target gene ELK1. Furthermore, the lncRNA ODSM partially reduced the apoptosis and promoted the differentiation of MC3T3-E1 cells in a MG unloading environment, and these effects were partly dependent on miR-139-3p. Targeted overexpression of the lncRNA ODSM in HU mice could regulate the number and function of osteoblasts while partially reversing bone loss caused by mechanical unloading. Our research revealed the function of the lncRNA ODSM in osteoblasts and is the first to indicate the promising value of the lncRNA ODSM in the preventative treatment of bone loss caused by mechanical unloading.

## Materials and methods

### Cell culture

The mouse preosteoblast MC3T3-E1 cell line was purchased from the Cell Bank of the Chinese Academy of Sciences (Shanghai, China). Under standard cell culture conditions of 5% CO_2_, 95% humidity, and 37 °C, 10% fetal bovine serum (HyClone, USA) and 1% penicillin/streptomycin (HyClone, USA) were added to α-MEM (Gibco, USA). Eight to 12 generations of cells were used in the experiments, and cells used in experiments were in good condition without mycoplasma contamination. To study the function of osteogenic differentiation, MC3T3-E1 cells were induced with osteogenic medium supplemented with 100 nM dexamethasone, 10 mM β-glycerophosphate (Sigma, USA), and 50 μM ascorbic acid. All cell experiments were repeated three times with cells that were not resuscitated at the same time, and experimenter were aware of the grouping situation.

### Cell transfection

The sequences of the siRNAs and negative controls for the lncRNA ODSM are shown in Supplementary Table 1. The lncRNA ODSM (pEX-ODSM) vectors and negative controls were purchased from GenePharma (Shanghai, China). The concentration of transfected siRNA was 80 nM, and the plasmid concentration was 200 ng/μl. The concentration of miR-139-3p mimic and its negative control (RiboBio, China) used for transfection was 50 nM, and the concentration of the inhibitor and its negative control (RiboBio, China) used for transfection was 100 nM. The siRNAs, plasmids, and miRNA modulator oligomers were transfected into MC3T3-E1 cells using Lipofectamine 3000 (Invitrogen, USA).

### Flow cytometry

MC3T3-E1 cells were digested with 0.125% trypsin solution. After cells were washed with phosphate-buffered saline (PBS) and centrifuged for 5 min at 1000 rpm, they were resuspended in PBS and stained using an Annexin V-FITC Apoptosis Detection Kit (BioVision, USA). Apoptosis rates were analyzed by flow cytometry (BD Bioscience, USA).

### Western blotting analysis

MC3T3-E1 cells were collected into RIPA buffer (Thermo Scientific, USA). The same amount of protein samples was loaded onto NuPage Bis-Tris polyacrylamide gels (Invitrogen, USA), and proteins were then transferred to polyvinylidene difluoride membranes. Membranes were then blocked for 4 h with 5% milk at room temperature and cultured overnight at 4 °C with primary antibodies against the following specific proteins: Bax (1:1000; Cell Signaling Technology #2772, USA), Bcl-2 (1:1000; Cell Signaling Technology #3498, USA), caspase-3 (1:1000; Cell Signaling Technology #9662, USA), Runx2 (1:1000; Cell Signaling Technology #12556 S, USA), Bglap (1:500; Abcam ab93876, USA), Col1a1 (1:1000; Abcam ab34710, USA), ELK1 (1:500; Abcam ab131465, USA), and GAPDH (1:5000; Proteintech 60004-1-Ig, USA). Next, membranes were incubated with peroxidase-conjugated secondary antibody (1:5000; Jackson, USA), and signals were visualized by Super Signal West substrate (Thermo Fisher Scientific, USA). Band densities were measured and analyzed by ImageJ software.

### Hoechst staining

A Hoechst staining kit (Beyotime Biotechnology, China) was used to detect apoptosis in MC3T3-E1 cells according to the manufacturer’s instructions. Briefly, cells were stained with Hoechst 33258 staining solution after fixation in 4% paraformaldehyde. Then, the fluorescence intensity of the stained cells was measured by an Olympus fluorescence microscope (Olympus Corporation, Japan).

### Alizarin red staining

MC3T3-E1 cells were seeded into 12-well plates and cultured in osteogenic medium for 21 days. Cells were fixed in 70% cold ethanol for 1 h and rinsed gently with ddH_2_O. Then, osteoblasts were stained using 40 mM Alizarin red S (Sigma-Aldrich, Missouri, USA) at a pH of 4.2 for 15 min and rinsed again gently with ddH_2_O three times. After rinsing with Dulbecco's PBS for 15 min, representative images were acquired using a digital camera.

### RNA-FISH

RNA-FISH was used to detect the localization of lncRNA ODSM and miR-139-3p in MC3T3-E1 cells. After immobilization with 4% paraformaldehyde for 20 min at room temperature, the cells were prehybridized with a hybridization solution. Then, lncRNA ODSM and miR-139-3p were labeled with CY3 fluorophore and FAM fluorophore, respectively. Finally, 4′,6-diamidino-2-phenylindole (DAPI) was used to stain the nuclei, and fluorescence images were taken using an FV1000 confocal microscope (Olympus, Japan).

### Immunofluorescence

MC3T3-E1 cells were rinsed gently with PBS and fixed with 4% paraformaldehyde for 15 min. Then, cells were permeabilized with 0.025% Triton X-100 for 10 min. Cells were incubated with 1% normal goat serum for 1 h and incubated overnight at 4 °C with anti-ELK1 primary antibody (1:100; Abcam ab131465, USA). Next, cells were rinsed three times and incubated with a fluorescein isothiocyanate (FITC)-conjugated secondary antibody (Abcam, USA) for 1 h. Finally, cells were stained with DAPI for 10 min at room temperature. Representative images were acquired using an FV1000 confocal microscope (Olympus, Japan).

### Cell culture under MG unloading conditions

2D clinorotation (developed by the China Astronaut Research and Training Center, Beijing) can provide an unloading condition for cells cultured in a simulate MG environment. Experiments were carried out according to the previous description^25^. MC3T3-E1 cells were seeded on coverslips. MC3T3-E1 cells were plated on the cover glasses in a six-well plate at a density of 1 × 10^5^ cells per well. After ~8 h, cells adhered to the walls, and the cover glasses were inserted into a chamber filled with culture medium. The distance between the cover glasses and the rotating axis of the chamber was 12.5 mm. Then, the caps of the chamber were tightened after all bubbles were gently removed. Finally, the chambers were placed into a clinostat and rotated around a horizontal axis at 24 rpm. The vertical rotation group served as the control group. The whole process of cell culture in the MG unloading environment was carried out at 37 °C.

### qRT-PCR analysis

Total RNA was extracted from cells or bone tissues using RNAiso Plus (TaKaRa, Japan), and cDNA was synthesized by using a PrimeScript RT Master Mix reagent kit (TaKaRa, Japan). Subsequent real-time PCR was performed using SYBR^®^ Premix Ex Taq^TM^ II (TaKaRa, Japan) and a CFX96 real-time PCR detection system (BIO-RAD, USA). GAPDH was used as the reference gene, and U6 was used as the endogenous control for normalization. The primers used for real-time PCR are listed in Supplementary Table 1.

### ALP activity assay

MC3T3-E1 cells were harvested in mammalian protein extraction reagent (Pierce, USA), and supernatants were collected after centrifugation at 12,000 × *g* for 15 min. We used an ALP assay kit (Nanjing Jiancheng Technological Inc., China) to measure ALP activity and a BCA protein assay kit (Thermo Fisher Scientific, USA) to quantify the cellular protein concentration. ALP activity (IU/L) was determined as the production of 1 nmol of p-nitrophenol from 1 µg of total cellular protein in 1 min.

### ALP staining

After culture in osteogenic medium for 7 days, MC3T3-E1 cells were stained using an NBT/BCIP staining kit (Beyotime Biotechnology, China) according to the manufacturer’s protocol. Staining for each group was repeated at least three times, and representative images were acquired using a digital camera.

### Luciferase assay

The ELK1 3′-UTR with the wild-type miR-139-3p binding site was generated as described previously, and the sequences were inserted into the pmirGLO luciferase vector (Promega, USA). The 293 T cells with low endogenous miRNA expression were selected, and luciferase reporter plasmids were transfected into these cells. Then, 293 T cells were cotransfected with miR-139-3p (mimic-139 or its negative control) and lncRNA ODSM vector (pEX-ODSM WT, pEX-ODSM MUT, or their negative control) using Lipofectamine 3000 (Invitrogen, USA). Finally, the firefly and Renilla luciferase activities were measured using a dual luciferase assay system (Promega, USA).

### HU model

The hindlimb unloading model is one of the models of bone loss caused by mechanical unloading. Six-month-old male C57BL/6 J mice were maintained under standard conditions (12 h light/12 h dark cycle, 21 °C controlled temperature). Twenty mice were randomly divided into four groups by random scale, as follows: (1) Con, (2) HU, (3) HU + pEX, and (4) HU + pEX-ODSM (*N* *=* 5). The HU mice were hung from the top of the cage by the tail at a 30° angle with only the forelimbs touching the floor, which allowed them to move and access food and water freely. Before hindlimb unloading, mice in the experimental groups (HU + pEX and HU + pEX-ODSM) were injected with 2 mg/kg plasmids every day for three consecutive days. The pEX-ODSM (or the negative control pEX) vectors and the (AspSerSer)_6_-liposome delivery systems were utilized as described previously^31^. After 3 weeks of tail suspension, all mice were in normal condition without abnormal death. Mice were euthanized, and the bilateral femurs and tibiae were harvested. In animal experiments, the researchers who took samples and tested the indicators did not know the grouping situation. These study procedures were approved by the Air Force Medical University Animal Ethics and Experimental Safety Committee.

### Histology

Harvested femurs were fixed in 4% paraformaldehyde, decalcified in 10% ethylenediaminetetraacetic acid (Beyotime Biotechnology, Shanghai, China), and embedded in paraffin. For histological analysis, bone sections were stained with H&E according to the manufacturer’s protocol (Sigma, USA). For immunohistochemistry, the slices were dewaxed in water and immersed in 5% goat serum and were then incubated overnight at 4 °C with primary antibodies against the following specific proteins: ELK1 (1:50; Abcam ab131465, USA) and Bglap (1:50; Abcam ab93876, USA). Subsequently, diaminobenzidine and hematoxylin were used to detect immunoreactivity. To evaluate the dynamic indexes of bone formation, mice were subcutaneously injected with calcein (Sigma, USA, 8 mg/kg) on the tenth and third days before euthanasia. After embedding and sectioning of hard tissue, an FV1000 confocal microscope (Olympus, Japan) was used for observation and recording. Bone dynamic histomorphometric analysis of the MAR was performed to assess calcium deposition.

### TUNEL assay

After tibias were dissected and fixed in 4% paraformaldehyde, they were decalcified and embedded according to the sample preparation protocol for high-resolution 3D confocal imaging of mouse skeletal tissue^46^. Then, slides were stained with the DeadEnd™ Fluorometric TUNEL System (Promega, USA). Apoptotic cell quantification was performed by counting the TUNEL-positive nuclei in Olympus cellSens Standard software.

### microCT analysis

Each mouse femur was fixed in 4% paraformaldehyde for 24 h and scanned by a microCT scanner (Siemens, Germany) with energy of 80 kV and 500 mA. Femurs were scanned over a total angle of 360° at incremental angles of 0.5 degrees. The scanning time was 800 ms/frame at a resolution of 10.44 μm. The region of interest (ROI) represents the microstructure of the femur, which was 1500 μm above the proximal epiphyseal growth plate and was selected as a 2.5 × 2.5 × 3 mm^3^ cube. The parameters, including BMD, BV/TV, Tb.Th, Tb.N, BS/BV, Tb.Sp, and TbPF were analyzed by COBRA software for microCT. These data were collected for blinded analyses.

### Three-point bending test

A three-point bending test was performed to measure bone strength by using an electromechanical material testing machine (Bose, Massachusetts, USA). Femoral specimens from mice were evaluated under a microscope to ensure that the cortex was intact, and femurs were kept wet during the test. Femurs were placed on the bending fixture with the short axis of the femur aligned with the direction of the force. The span of the two fulcrums was 8 mm, the preload was 0.5 N, and the loading speed was 0.02 mm/s. After the femurs were destroyed, the test was terminated. The parameters of load-deflection curves—maximum load at failure (N), stiffness (the slope of the load-deflection curve, representing the elastic deformation, N/mm), and modulus of elasticity (Gpa)—were calculated according to Turner et al.^47^.

### Statistical analysis

All statistical analyses were performed using SPSS 22.0 software. All numerical data are shown as the means ± SDs from at least three or five duplicate experiments. Statistical significance was assessed by a two-tailed *t*-test or one-way analysis of variance. A *P*-value of <0.05 was considered to be significant.

## Supplementary information

Supplementary figure legends

Supplementary Table 1

Supplementary figure 1

Supplementary figure 2

Supplementary figure 3

Supplementary figure 4

Supplementary figure 5

## References

[1] Florencio-Silva R, Sasso GR, Sasso-Cerri E, Simões MJ, Cerri PS (2015). Biology of bone tissue: structure, function, and factors that influence bone cells. Biomed. Res. Int..

[2] Robling AG, Castillo AB, Turner CH (2006). Biomechanical and molecular regulation of bone remodeling. Annu. Rev. Biomed. Eng..

[3] Clarke BL, Khosla S (2010). Physiology of bone loss. Radiol. Clin. North Am..

[4] Pietschmann P, Mechtcheriakova D, Meshcheryakova A, Foger-Samwald U, Ellinger I (2015). Immunology of Osteoporosis: a mini-review. Gerontology.

[5] Nagaraja MP, Risin D (2013). The current state of bone loss research: data from spaceflight and microgravity simulators. J. Cell. Biochem..

[6] Morse LR (2012). Association between sclerostin and bone density in chronic spinal cord injury. J. Bone Miner. Res..

[7] Stein EM (2013). Bariatric surgery results in cortical bone loss. J. Clin. Endocrinol. Metab..

[8] Morey-Holton ER, Globus RK (2002). Hindlimb unloading rodent model: technical aspects. J. Appl. Physiol..

[9] Grimm D (2016). The impact of microgravity on bone in humans. Bone.

[10] Shi W (2017). Microgravity induces inhibition of osteoblastic differentiation and mineralization through abrogating primary cilia. Sci. Rep..

[11] Caillot-Augusseau A (1998). Bone formation and resorption biological markers in cosmonauts during and after a 180-day space flight (Euromir 95). Clin. Chem..

[12] Arfat Y (2014). Physiological effects of microgravity on bone cells. Calcif. Tissue Int..

[13] Tsao YD, Goodwin TJ, Wolf DA, Spaulding GF (1992). Responses of gravity level variations on the NASA/JSC bioreactor system. Physiologist.

[14] Schwarz RP, Goodwin TJ, Wolf DA (1992). Cell culture for three-dimensional modeling in rotating-wall vessels: an application of simulated microgravity. J. Tissue Cult. Methods.

[15] Rinn JL, Chang HY (2012). Genome regulation by long noncoding RNAs. Annu. Rev. Biochem..

[16] Quinn JJ, Chang HY (2016). Unique features of long non-coding RNA biogenesis and function. Nat. Rev. Genet..

[17] Huynh NP, Anderson BA, Guilak F, McAlinden A (2017). Emerging roles for long noncoding RNAs in skeletal biology and disease. Connect. Tissue Res..

[18] Yin C (2019). A novel long noncoding RNA AK016739 inhibits osteoblast differentiation and bone formation. J. Cell. Physiol..

[19] Li CJ (2018). Long noncoding RNA Bmncr regulates mesenchymal stem cell fate during skeletal aging. J. Clin. Invest..

[20] He S (2019). LncRNA ODIR1 inhibits osteogenic differentiation of hUC-MSCs through the FBXO25/H2BK120ub/H3K4me3/OSX axis. Cell Death Dis..

[21] Thomson DW, Dinger ME (2016). Endogenous microRNA sponges: evidence and controversy. Nat. Rev. Genet..

[22] Salmena L, Poliseno L, Tay Y, Kats L, Pandolfi PP (2011). A ceRNA hypothesis: the Rosetta Stone of a hidden RNA language. Cell.

[23] Yuan H (2019). A novel long noncoding RNA PGC1β-OT1 regulates adipocyte and osteoblast differentiation through antagonizing miR-148a-3p. Cell Death Differ..

[24] Feng L (2018). Linc-ROR promotes osteogenic differentiation of mesenchymal stem cells by functioning as a competing endogenous RNA for miR-138 and miR-145. Mol. Ther. Nucleic Acids.

[25] Yang L (2019). The long non-coding RNA-ORLNC1 regulates bone mass by directing mesenchymal stem cell fate. Mol. Ther..

[26] Bu Y, Zheng D, Wang L, Liu J (2018). LncRNA TSIX promotes osteoblast apoptosis in particle-induced osteolysis by down-regulating miR-30a-5p. Connect. Tissue Res..

[27] Wang Y (2018). MicroRNA-139-3p regulates osteoblast differentiation and apoptosis by targeting ELK1 and interacting with long noncoding RNA ODSM. Cell Death Dis..

[28] Zhang ZK (2018). Long noncoding RNA lncMUMA reverses established skeletal muscle atrophy following mechanical unloading. Mol. Ther..

[29] Zhang ZK (2018). A newly identified lncRNA MAR1 acts as a miR-487b sponge to promote skeletal muscle differentiation and regeneration. J. Cachexia Sarcopenia Muscle.

[30] Wang X (2013). miR-214 targets ATF4 to inhibit bone formation. Nat. Med..

[31] Zhang G (2012). A delivery system targeting bone formation surfaces to facilitate RNAi-based anabolic therapy. Nat. Med..

[32] Wang H (2018). Osteoblast-targeted delivery of miR-33-5p attenuates osteopenia development induced by mechanical unloading in mice. Cell Death Dis..

[33] Arumugam B (2019). Parathyroid hormone-stimulation of Runx2 during osteoblast differentiation via the regulation of lnc-SUPT3H-1:16 (RUNX2-AS1:32) and miR-6797-5p. Biochimie.

[34] Alemdehy MF (2015). ICL-induced miR139-3p and miR199a-3p have opposite roles in hematopoietic cell expansion and leukemic transformation. Blood.

[35] Zou, Z. C. et al. MicroRNA-139-3p suppresses tumor growth and metastasis in hepatocellular carcinoma by repressing ANXA2R. *Oncol. Res*. **26**, 1391–1399 (2018). 10.3727/096504018X15178798885361 PMC7844686 29422116

[36] Yonemori M (2016). Dual tumor-suppressors miR-139-5p and miR-139-3p targeting matrix metalloprotease 11 in bladder cancer. Cancer Sci..

[37] Long H (2017). miR-139-5p represses bmsc osteogenesis via targeting Wnt/β-catenin signaling pathway. DNA Cell Biol..

[38] Sheng K, Lu J, Zhao H (2018). ELK1-induced upregulation of lncRNA HOXA10-AS promotes lung adenocarcinoma progression by increasing Wnt/β-catenin signaling. Biochem. Biophys. Res. Commun..

[39] Zhang Y (2009). Co-stimulation of the bone-related Runx2 P1 promoter in mesenchymal cells by SP1 and ETS transcription factors at polymorphic purine-rich DNA sequences (Y-repeats). J. Biol. Chem..

[40] Kim HK, Kim MG, Leem KH (2014). Collagen hydrolysates increased osteogenic gene expressions via a MAPK signaling pathway in MG-63 human osteoblasts. Food Funct..

[41] Wu CC (2006). Roles of MAP kinases in the regulation of bone matrix gene expressions in human osteoblasts by oscillatory fluid flow. J. Cell. Biochem..

[42] Ying W (2016). miR-150 regulates obesity-associated insulin resistance by controlling B cell functions. Sci. Rep..

[43] Jin X (2017). LncRNA-TCONS_00026907 is involved in the progression and prognosis of cervical cancer through inhibiting miR-143-5p. Cancer Med..

[44] Liang C (2018). Inhibition of osteoblastic Smurf1 promotes bone formation in mouse models of distinctive age-related osteoporosis. Nat. Commun..

[45] Liu J (2017). Increased PLEKHO1 within osteoblasts suppresses Smad-dependent BMP signaling to inhibit bone formation during aging. Aging Cell.

[46] Kusumbe AP, Ramasamy SK, Starsichova A, Adams RH (2015). Sample preparation for high-resolution 3D confocal imaging of mouse skeletal tissue. Nat. Protoc..

[47] Turner CH, Burr DB (1993). Basic biomechanical measurements of bone: a tutorial. Bone.

