# Supplementary material for: Targeted overexpression of the long noncoding RNA ODSM can regulate osteoblast function in vitro and in vivo
Source: Cell Death Dis. 2020 Feb 18;11(2):133. doi: 10.1038/s41419-020-2325-3 (PMC7028725; doi:10.1038/s41419-020-2325-3)
Supplement: Supplementary file 1 — Supplementary figure legends [file 41419_2020_2325_MOESM1_ESM.docx]

**Supplementary Fig 1.** **LncRNA ODSM decreased in mice femurs after HU for 21 days.** (a) Real-time PCR analysis of lncRNA ODSM levels in different tissues and organs of mice. (*N*=5) **P <* 0.05 vs. femur. (b) Real-time PCR analysis of lncRNA ODSM levels in femurs from normal and HU mice. (*N*=5) **P <* 0.05 vs. control.

**Supplementary Fig 2.** **LncRNA ODSM primarily localizes in the cytoplasm in MC3T3-E1 cells.** (a) Presentation of score of lncRNA ODSM at different subcellular locations by lncLocator (http://www.csbio.sjtu.edu.cn/bioinf/lncLocator/). (b) Real-time PCR analysis of lncRNA ODSM expression in the cytoplasmic (Cyt) and nuclear (Nuc) fractions. The control, 12s rRNA was found primarily in the cytoplasmic fraction and 45s ribosomal RNA (rRNA) was primarily localized in the nucleus (*N*=3). **P <* 0.05

**Supplementary Fig 3.** **LncRNA ODSM regulates ELK1 and pELK1 expressions in MC3T3-E1 cells.** (a, b) Protein levels of ELK1 and pELK1 in osteoblasts (*N*=3). **P <* 0.05 *vs*. negative control.

**Supplementary Fig 4.** **LncRNA ODSM interacts with miR-139-3p to regulate ELK1 expression.** (a) Protein levels of ELK1 in osteoblasts (*N*=3). (b) Immunostaining analysis of the expression levels of ELK1 in osteoblasts (*N*=3). Scale bar, 50 µm. **P* < 0.05, ***P* < 0.01 vs. the negative control.

**Supplementary Fig 5.** **Expression changes of lncRNA ODSM and miR-139-3p in mice femurs** **after HU for 21 days.** (a, b) qRT-PCR analysis of lncRNA ODSM and miR-139-3p levels in femurs of mice from each group after HU for 21 days. (*N*=3) **P <* 0.05, ***P <* 0.01 vs. control or negative control.
