# Supplementary material for: Targeted overexpression of the long noncoding RNA ODSM can regulate osteoblast function in vitro and in vivo
Source: Cell Death Dis. 2020 Feb 18;11(2):133. doi: 10.1038/s41419-020-2325-3 (PMC7028725; doi:10.1038/s41419-020-2325-3)
Supplement: Supplementary file 2 — Supplementary Table 1 [file 41419_2020_2325_MOESM2_ESM.docx]

**Supplement Table 1. The sequence of primers and siRNAs.**

| Name | Sequence (5'-3') |
| --- | --- |
| siR-ODSM sense | GCU CUC UCC CUG ACU GUU ATT |
| siR-ODSM antisense | UAA CAG UCA GGG AGA GAG CTT |
| siR-NC sense | UUC UCC GAA CGU GUC ACG UTT |
| siR-NC antisense | ACG UGA CAC GUU CGG AGA ATT |
| LncRNA ODSM-F | GCA AAG TTG TGC CAT CCA G |
| LncRNA ODSM-R | CCA CTT AGC GAT AAA AAG AAA TCT |
| Runx2-F | GAA CCA AGA AGG CAC AGA CAG A |
| Runx2-R | GGC GGG ACA CCT ACT CTC ATA C |
| ALP-F | GCA GTA TGA ATT GAA TCG GAA CAA C |
| ALP-R | ATG GCC TGG TCC ATC TCC AC |
| Bglap-F | GAC CGC CTA CAA ACG CAT CTA |
| Bglap-R | CAG AGA GAG AGG ACA GGG AGG A |
| Col1a1-F | GAC ATG TTC AGC TTT GTG GAC CTC |
| Col1a1-R | GGG ACC CTT AGG CCA TTG TGT A |
| GAPDH-F | TGT CCG TCG TGG ATC TGA |
| GAPDH-R | TTG CTG TTG AAG TCG CAG GAG |
