# Supplementary figures and images for: Targeted overexpression of the long noncoding RNA ODSM can regulate osteoblast function in vitro and in vivo
Source: Cell Death Dis. 2020 Feb 18;11(2):133. doi: 10.1038/s41419-020-2325-3 (PMC7028725; doi:10.1038/s41419-020-2325-3)

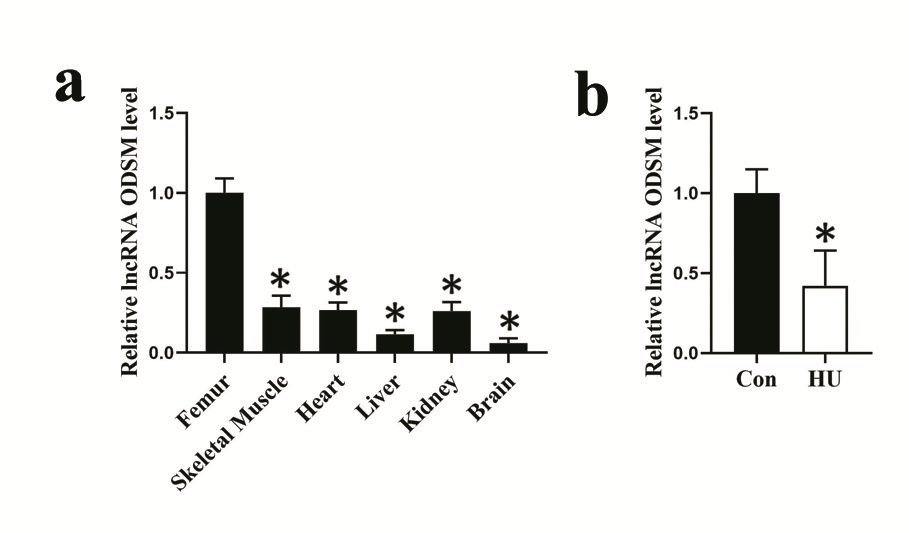

Supplement: Supplementary file 3 — Supplementary figure 1 [file 41419_2020_2325_MOESM3_ESM.tif]

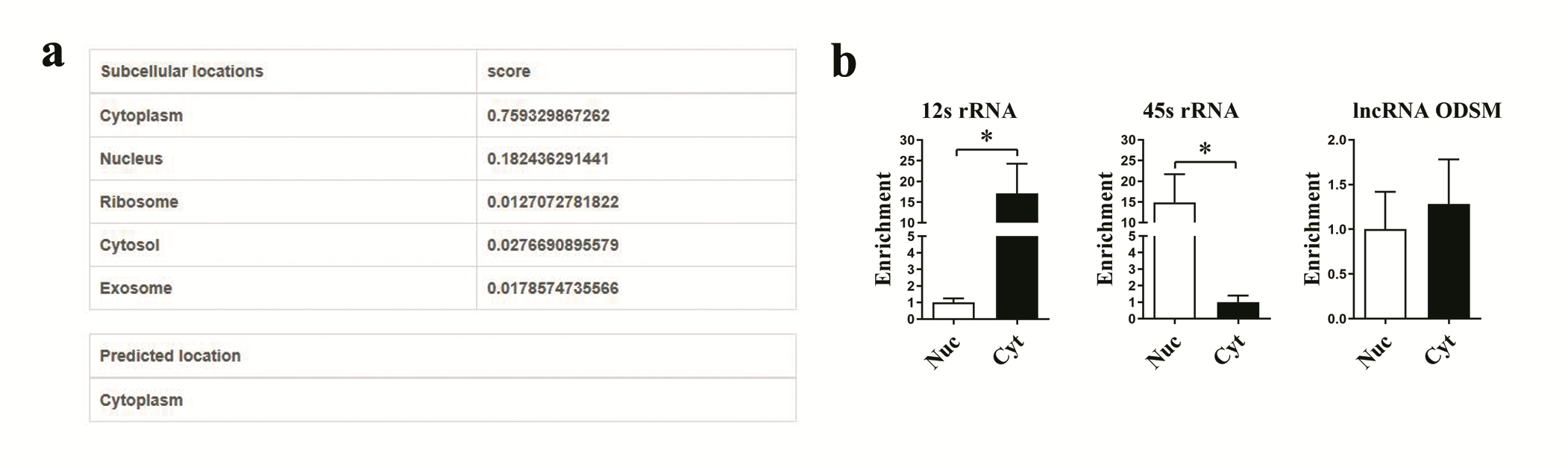

Supplement: Supplementary file 4 — Supplementary figure 2 [file 41419_2020_2325_MOESM4_ESM.tif]

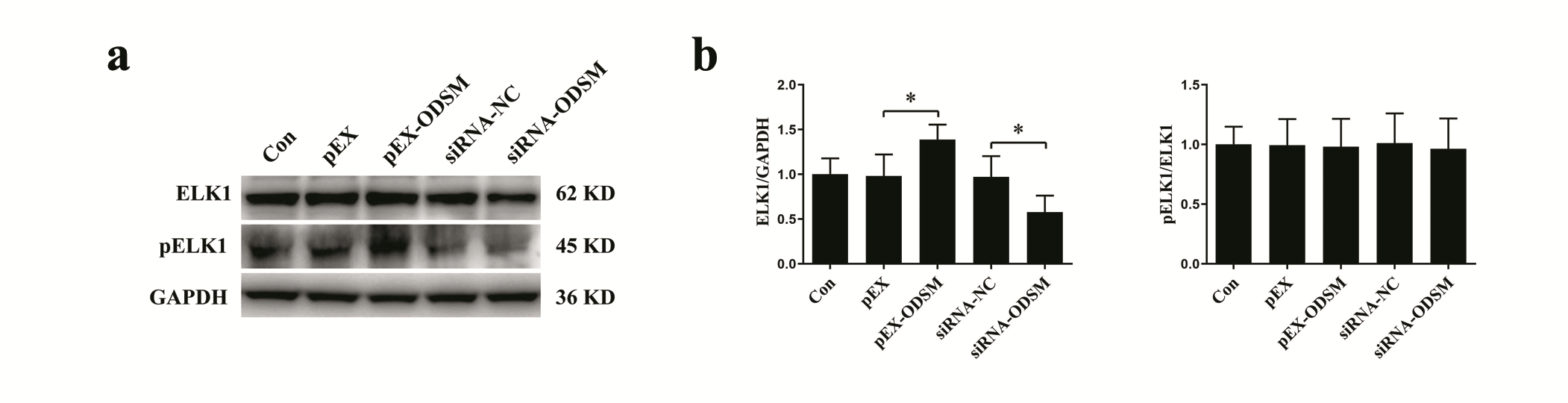

Supplement: Supplementary file 5 — Supplementary figure 3 [file 41419_2020_2325_MOESM5_ESM.tif]

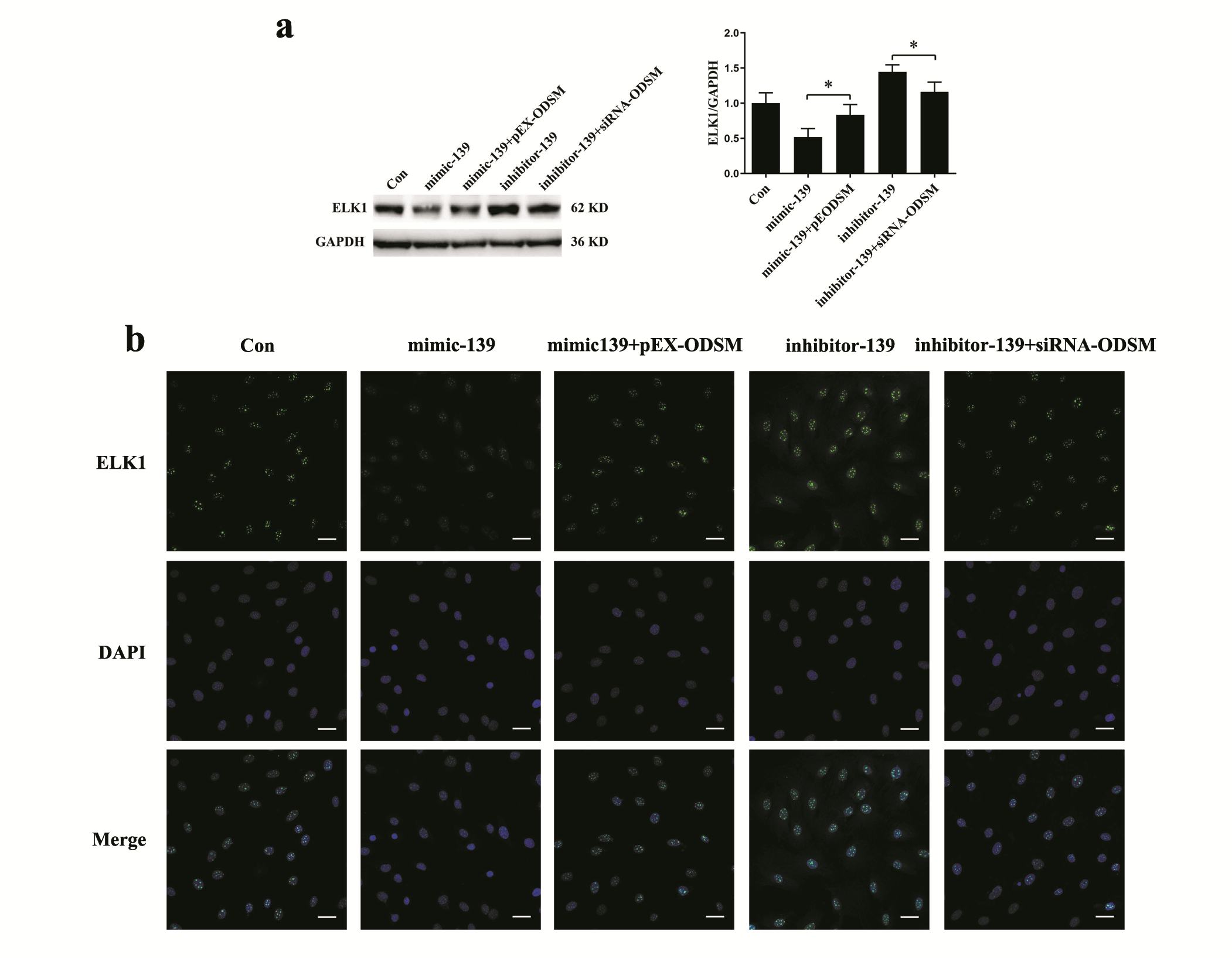

Supplement: Supplementary file 6 — Supplementary figure 4 [file 41419_2020_2325_MOESM6_ESM.tif]

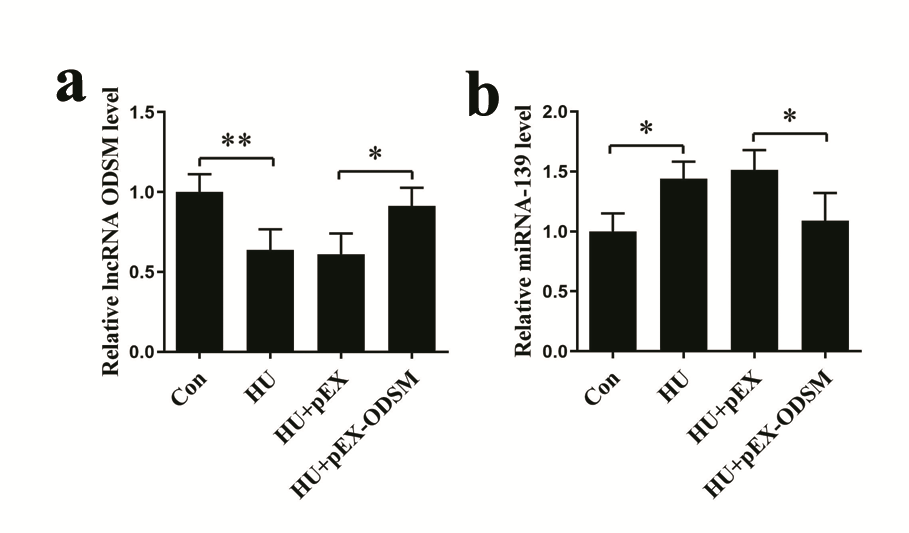

Supplement: Supplementary file 7 — Supplementary figure 5 [file 41419_2020_2325_MOESM7_ESM.tif]
